# Supplementary material for: Defining Signatures of Arm-Wise Copy Number Change and Their Associated Drivers in Kidney Cancers
Source: Int J Mol Sci. 2019 Nov 16;20(22):5762. doi: 10.3390/ijms20225762 (PMC6887958; doi:10.3390/ijms20225762)
Supplement: Supplementary file 1 [file ijms-20-05762-s001.zip › supplementary figures.pdf]

## Supplementary Figures

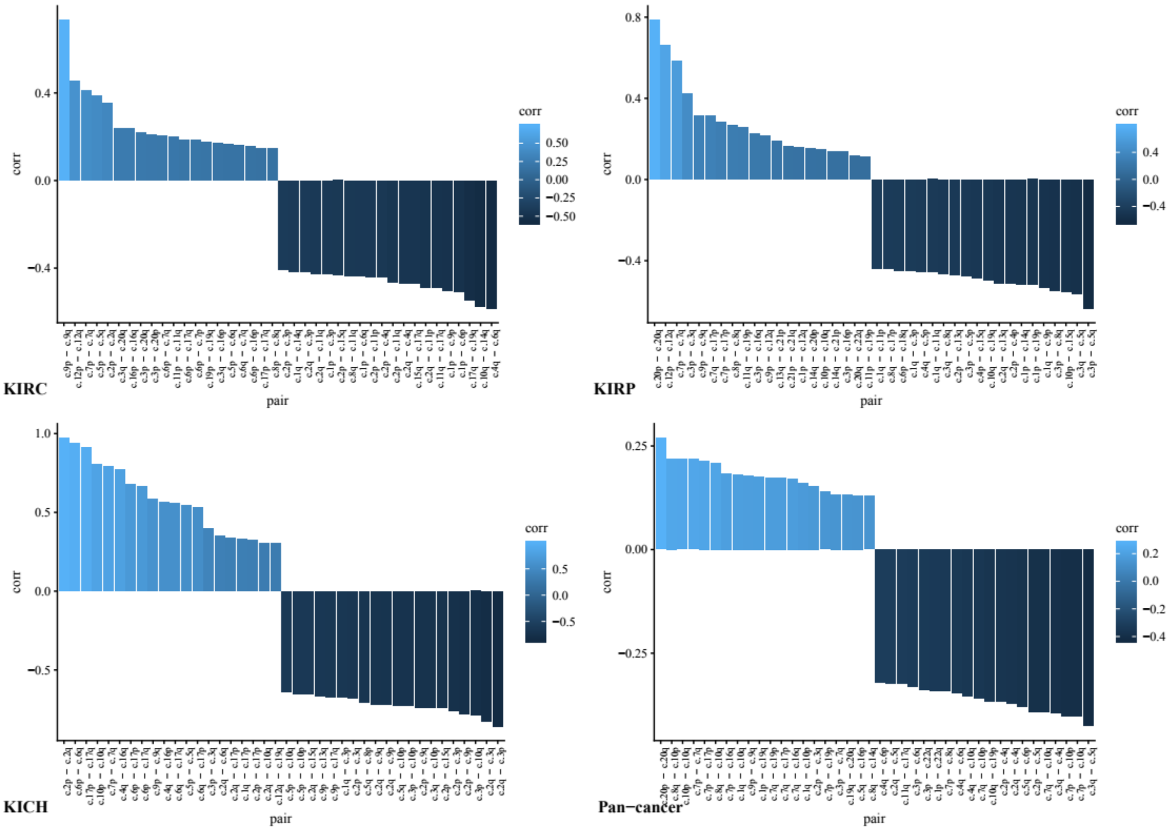


**Supplementary Figure 1.** A ranked bar chart showing the most extreme arm-wise copy number correlation coefficients.

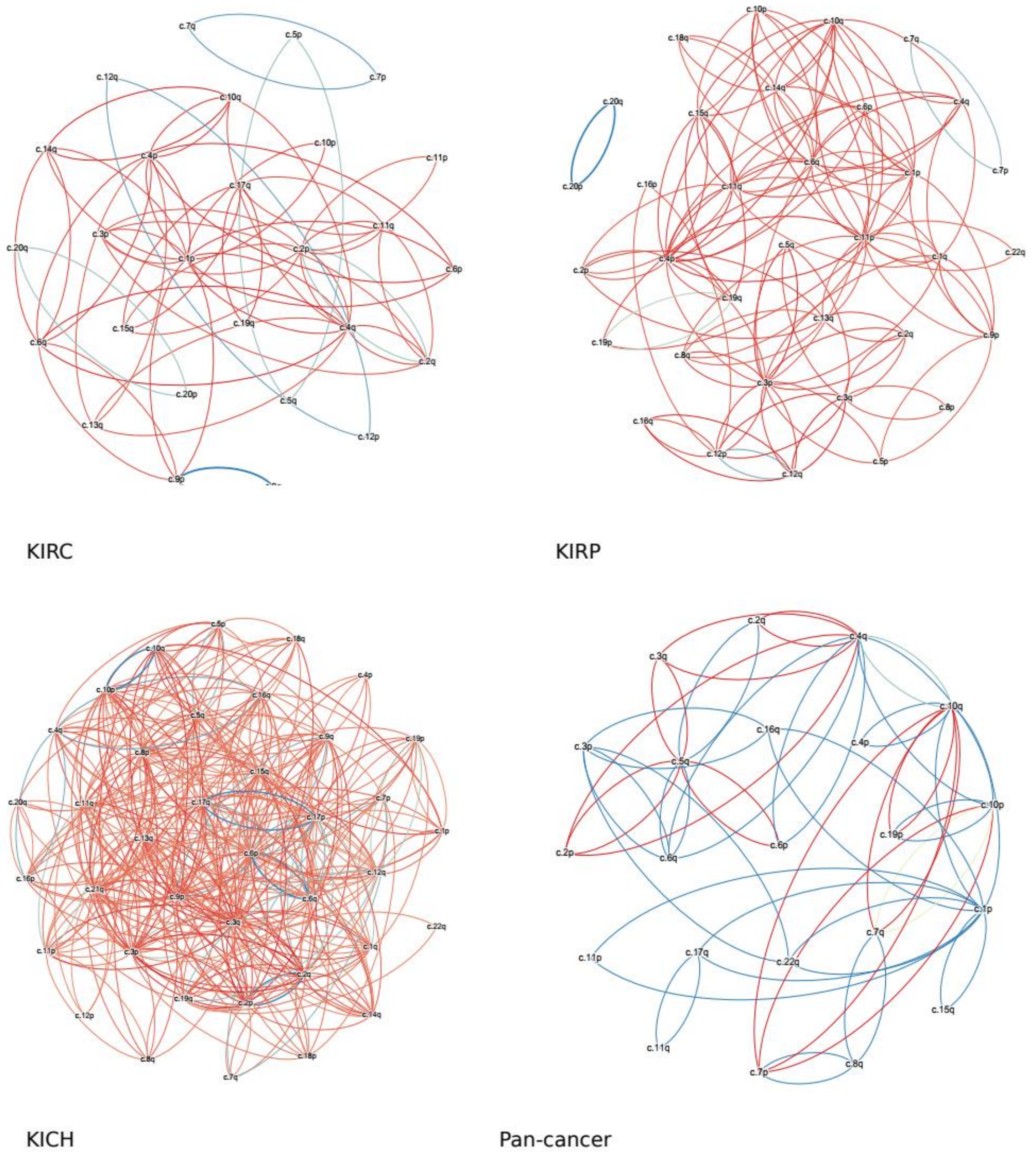


**Supplementary Figure 2.** A graphical illustration of arm-wise copy number correlation. Nodes represent chromosome arms and edges represent correlations of above a threshold of r=0.4 and below r=-0.4. Red edges denote positive correlation between the chromosome arms and blue represent negative correlations.
